# Obstructive Sleep Apnoea Syndrome, Endothelial Function and Markers of Endothelialization. Changes after CPAP

**DOI:** 10.1371/journal.pone.0122091

**Published:** 2015-03-27

**Authors:** Rocio Muñoz-Hernandez, Antonio J. Vallejo-Vaz, Angeles Sanchez Armengol, Rafael Moreno-Luna, Candela Caballero-Eraso, Hada C. Macher, Jose Villar, Ana M Merino, Javier Castell, Francisco Capote, Pablo Stiefel

**Affiliations:** 1 Laboratorio de Hipertensión Arterial e Hipercolesterolemia, Instituto de Biomedicina de Sevilla (IBiS), Hospital Universitario Virgen del Rocío/CSIC/Universidad de Sevilla, Sevilla, Spain; 2 Unidad del Sueño, Unidad Medico Quirúrgica de Enfermedades Respiratorias, Hospital Virgen del Rocío, Sevilla, Spain; 3 Departamento de Fisiopatología Vascular, Hospital Nacional de Parapléjicos, SESCAM, Toledo, Spain; 4 Servicio de Bioquímica Clínica, Hospital Virgen del Rocío, Sevilla, Spain; 5 Unidad Clínico Experimental de Riesgo Vascular (UCAMI), Hospital Virgen del Rocío, Sevilla, Spain; 6 Instituto de Investigación Biomédica Bellvitge (IDIBELL), L'Hospitalet de Llobregat, Barcelona, Spain; 7 UGC de Radiología, Hospital Virgen del Rocío, Sevilla, Spain; The Chinese University of Hong Kong, HONG KONG

## Abstract

**Study objectives:**

This study tries to assess the endothelial function in vivo using flow-mediated dilatation (FMD) and several biomarkers of endothelium formation/restoration and damage in patients with obstructive sleep apnoea (OSA) syndrome at baseline and after three months with CPAP therapy.

**Design:**

Observational study, before and after CPAP therapy.

**Setting and Patients:**

We studied 30 patients with apnoea/hypopnoea index (AHI) >15/h that were compared with themselves after three months of CPAP therapy. FMD was assessed non-invasively in vivo using the Laser-Doppler flowmetry. Circulating cell-free DNA (cf-DNA) and microparticles (MPs) were measured as markers of endothelial damage and the vascular endothelial growth factor (VEGF) was determined as a marker of endothelial restoration process.

**Measurements and results:**

After three month with CPAP, FMD significantly increased (1072.26 ± 483.21 vs. 1604.38 ± 915.69 PU, p< 0.005) cf-DNA and MPs significantly decreased (187.93 ± 115.81 vs. 121.28 ± 78.98 pg/ml, p<0.01, and 69.60 ± 62.60 vs. 39.82 ± 22.14 U/μL, p<0.05, respectively) and VEGF levels increased (585.02 ± 246.06 vs. 641.11 ± 212.69 pg/ml, p<0.05). These changes were higher in patients with more severe disease. There was a relationship between markers of damage (r = -0.53, p<0.005) but not between markers of damage and restoration, thus suggesting that both types of markers should be measured together.

**Conclusions:**

CPAP therapy improves FMD. This improvement may be related to an increase of endothelial restoration process and a decrease of endothelial damage.

## Introduction

Obstructive sleep apnoea (OSA) syndrome is a respiratory disorder characterized by the presence of apnoeas-hypopneas during sleep that lead to episodes of intermittent hypoxia. A relationship among OSA, vascular risk factors and vascular disease has been described in large prospective studies [1,2]. However, the underlying mechanisms linking OSA syndrome and vascular pathology remain unclear. Several studies have suggested that patients with OSA syndrome have an endothelial dysfunction [3,4] related to the intermittent hypoxemia and consequent generation of reactive oxygen species (ROS), pro-inflammatory molecules, markers of increased oxidative stress, as well as several coagulation and lipid metabolism disorders [5]. Interestingly, treatment of the intermittent hypoxemia with continuous positive airway pressure (CPAP) leads to an improvement in endothelial function [6].

Endothelial dysfunction can be assessed by measuring several biomarkers in plasma, but also by assessing endothelial function in vivo measuring flow-mediated dilatation (FMD) using high-definition ultrasonography [7] or Laser-Doppler flowmetry [8]. More recently, new markers of endothelial development and damage have been suggested. The production and release of pro-angiogenic factors such as the vascular endothelial growth factor (VEGF) can mobilize endothelial progenitor cells and may enhance the recruitment of these cells to the injured vascular tissue [9]. Conversely, circulating microparticles (MPs) are small vesicles (<1 μm) released from the endothelium in response to several injuries and that have been reported to be increased in cerebrovascular disease, hypertension, diabetes, smoking and coronary artery disease [5]. Recent evidence suggest that CD31^+^/annexin V^+^ MPs are increased in conditions of systemic endothelial cell damage and cardiovascular disease [10]. Finally, circulating cell-free DNA (cf-DNA) is considered as another marker of cell damage measurable in the bloodstream that may increase in different situations involving hypoxia [11].

Our purpose in this study was to assess the endothelial function in vivo, using FMD by Laser-Doppler flowmetry, and to measure different markers of endothelium formation and damage (VEGF,CD31^+^/annexin V^+^ MPs and circulating cf-DNA) in a group of patients diagnosed with OSA, at baseline and after three month with CPAP therapy. As a secondary objective we aimed to correlate the changes observed in those parameters with the severity of OSA syndrome at baseline.

## Materials and Methods

### Subjects

We included 30 consecutive patients diagnosed with OSA and having an apnoea-hypopnea index (AHI) greater than 15 per hour. Each patient was his own control after three months of CPAP therapy. Exclusion criteria were as follows: 1) absence of indication of CPAP, 2) prior treatment with CPAP, 3) refusal, intolerance, or less than four hours use of CPAP in the night-time, 4) any change of drugs that may have influence on blood pressure or endothelial function within the three months of follow-up, 5) glomerular filtration rate (MDRD) <30 ml/min/1,73m^2^. Evidence of any other acute or chronic condition that in the investigator’s opinion may have influence on the parameters assessed in our study was also considered for exclusion criteria.

This study was approved by the Human Research Review Committee at the Virgen del Rocío University Hospital and all the participants provided their written informed consent before inclusion.

### Polygraphy

All the included patients underwent attended respiratory polygraphy [“Sibelhome plus”® (Sibelmed, SIBEL S.A, Barcelona)] in the sleep laboratory of our center. Respiratory polygraphy included continuous recording of oronasal flow and pressure, heart rate, thoracic and abdominal respiratory movements, and oxygen saturation (SaO2). Polygraphy data were scored manually by trained personnel (the Spanish Society of Pulmonology and Thoracic Surgery guidelines were followed for the polygraphy readings [12]). Apnea was defined as an interruption of oronasal airflow for more than 10 seconds. Hypopnea was defined as a 50% reduction in oronasal airflow for more than 10 seconds associated with an oxygen desaturation of 4% or higher.

Apnea-hypopnea index (AHI) was defined as the number of apneas plus hypopneas per hour of recording, desaturation index was defined as the the number of oxygen desaturation per hour of recording. The average SaO2 was also measured.

### CPAP Pressure Titration

In the patients diagnosed of OSA, CPAP indication was performed according to the Spanish Society of Pulmonology and Thoracic Surgery guidelines [12]. In these patients, optimal CPAP pressure was titrated at home by an auto CPAP device (REMstar auto, Philips Respironics, Pennsylvania, USA) within a period of less than 15 days after the diagnostic study, according to a procedure previously validated [13]. The optimal fixed pressure was determined by trained personnel, based on the visual evaluation of the raw data recording from the night study.

### 24-hour- Ambulatory blood pressure monitoring

Blood pressure (BP) was measured using a validated 24-h device (SpaceLabs 90207, Redmond, WA, USA). It was programmed to measure BP every 20 min during 24 h. The limits to consider the diagnosis of hypertension and presence or absence of dipper pattern were those proposed in the ESC/ESH guidelines [14].

### Assessment of flow-mediated dilation (FMD) by Laser-Doppler flowmetry

A Laser-Doppler linear Periflux System 5000 (Perimed SA) was used to measure FMD in the forearm at 15 cm from the wrist after 4 minutes of ischemia provoked in the arm with a BP cuff (for details see reference [8]).

### Measurement of VEGF

Levels of vascular endothelial growth factor (VEGF) were analyzed in serum by ELISA using the Quantikine VEGF ELISA Kit (R&D Systems, Minneapolis, MN).

### Circulating CD31+/annexin V+ Microparticles

Heparin-buffered blood samples were obtained and processed and immediately spun at 13,000 × *g* for 20 minutes to separate the platelets.

Platelet-poor plasma was incubated for 15 min with a monoclonal antibody against FITC-labeled anti-CD31 antibody (BD Pharmingen. BD Bioscience, CA), followed by incubation with PE-conjugated Annexin V kits according to the manufacturer’s instructions (BD Pharmingen, BD bioscience, CA). The negative control (zero value) was obtained using the isotype antibodies. Flow Count Calibrator beads (Beckman Coulter, Marseille, France) were added. Analyses were performed in a Coulter Cytomic FC 500 flow cytometer (Beckman Coulter) with CXP software (Beckman Coulter). Each sample was measured in triplicate and the mean was taken as the final result.

### Circulating cell-free DNA measurement (cf-DNA)

Once collected, blood samples were spun for 8 minutes at 3,500 rpm, and the serum was frozen at −80°C for later DNA extraction. DNA was extracted automatically from the stored serum samples (400 μl) using a Compact MagnaPure Instrument and the nucleic acid isolation MagNA Pure Compact Nucleic Acid Isolation Kit I (Roche Diagnostics, Basel, Switzerland), according to the Total Plasma NA 100 400 V3 1 protocol. The DNA was resuspended in a final volume of 50 μl of water, and the serum DNA template was amplified in a final volume of 20 μl using a real-time quantitative polymerase chain reaction (PCR) assay for the beta-globin gene on a Light-Cycler 480 Real-Time PCR instrument (Roche Diagnostics), according to the manufacturer’s instructions. The β-globin Taqman system uses the following primers: beta-globin-354F (5′- TG CAC CTG ACT CCT GAG GAG A-3′); beta-globin-455R (5′-CCT TGA TAC CAA CCT GCC CAG-3′); and a dual-labeled fluorescent probe beta-globin-402T (5′-(FAM) TCT GGC CAA GTT TCA ACT CTG CTC GCT (TAMRA)-3′). Amplification was carried out over 48 cycles at 95°C for 5 minutes and at 62°C for 20 minutes, and the final size of the amplicon was 102 base pairs.

### Statistical analysis

Quantitative data were expressed as mean ± SD. The Shapiro–Wilk test was used to assess normality of distributions. We compared the changes after 3 months of CPAP therapy with respect to the baseline values using Student’s t-test for paired samples in case of normality. When samples were not normally distributed, a Wilcoxon paired rank test was used. Pearson correlations were carried out to explore the possible linear relationship between variables at baseline and changes after the CPAP-treatment period. Differences were considered to be significant when p values were less than 0.05. All analyses were performed with SSPS 15.0 software.

## Results

The baseline characteristics of the studied patients are shown in Table 1. The average compliance with CPAP therapy was 5.26 ± 1.61 hours/night. Table 2 illustrates the changes observed in several variables after the 3-month period with CPAP treatment. Briefly, BP decreased, particularly during the night-time, thus causing an increase in the number of subjects having a “dipper” pattern.

**Table 1:** Baseline characteristics of the patients.

**Age (years)**	51.70 ± 11.54 (95%CI: 47.39, 56.01)
**Sex (Male/Female)**	M: 19 (63.3%) / F: 11 (36.7%)
**Epworth's Questionnaire (points)**	10.73 ± 3.69 (95%CI: 9.35, 12.11)
**Apnoea-hypopnea index (No/h)**	56.28 ± 25.53 (95%CI: 46.74, 65.82)
**Oxygen desaturation Index**	54.49 ± 24.84 (95%CI: 45.21, 63.76)
**Mean oxygen saturation (%)**	90.57 ± 4.47 (95%CI: 88.9, 92.24)
**Arterial Hypertension**	17 (56.7%)
**Type 2 Diabetes Mellitus**	7 (23.3%)
**Hypercholesterolemia**	17 (56.7%)
**Current smokers**	9 (30.0%)
**Ex-smokers**	8 (26.7%)
**Coronary artery disease**	1 (3.3%)
**Ictus**	1 (3.3%)
**Body mass index (kg/m** ^2^)	35.83 ± 6.56 (95%CI: 33.38, 38.28)
**Waist circumference (cm)**	115.21 ± 12.53 (95%CI: 110.53, 119.89)
**Metabolic syndrome (ATP-III criteria)**	16 (53.3%)
**Mean number of Metabolic Syndrome criteria (ATP-III)**	2.66 ± 1.12 (95%CI: 2.24, 3.08)

Data are shown as mean ± SD (95% confidence intervals) or n (%).

**Table 2:** Changes of blood pressure, flow-mediated dilation, CD31^+^/annexin V^+^microparticles, circulating cell-free DNA and VEGF after CPAP.

	Baseline	After 3 months with CPAP therapy	p
**24 h SBP (mmHg)**	125.36 ± 12.28	121.27 ± 12.64	< 0.01
**24 h DBP (mmHg)**	76.06 ± 10.54	72.58 ± 10.93	< 0.005
**Day-time SBP (mmHg)**	128.20 ± 12.74	125.58 ± 14.20	< 0.05
**Day-time DBP (mmHg)**	79.13 ± 11.18	76.10 ± 12.15	< 0.005
**Night-time SBP (mmHg)**	118.24 ± 14.59	111.44 ± 11.95	< 0.005
**Night-time DBP (mmHg)**	68.62 ± 11.95	64.65 ± 10.53	< 0.05
**24 h. PP (mm Hg)** *	49.30 ± 7.61	48.68 ± 7.56	NS
**Day-time PP (mm Hg)** *	49.06 ± 8.21	49.48 ± 8.42	NS
**Night-time PP (mm Hg)** *	49.62 ± 7.58	46.79 ± 7.04	< 0.005
**AR-SBP (%)**	35.81 ± 25.95	27.11 ± 25.14	NS
**AR-DBP (%)**	32,52 ± 28,02	25,63 ± 27,08	< 0.05
**Dipper (%)**	20.7%	55.2%	< 0.05
**Non-Dipper (%)**	79.,3%	44.8%	<0.05
**FMD, hyperemic area (PU)**	1072.2 ± 483.2	1604.3 ± 915.6	< 0.005
**CD31** ^+^ **/Annexin V** ^+^ **MPs (U/μL)**	69.60 ± 62.60	39.82 ± 22.14	< 0.05
**cf-DNA (ng/ml)**	187.93 ± 115.81	121.28 ± 78.98	< 0.01
**VEGF (pg/ml)**	585.02 ± 246.06	641.11 ± 212.69	< 0.05

Data are shown as mean ± SD (95% confidence intervals) or n (%).

(*) Pulse pressure (PP) is defined as systolic minus diastolic blood pressure. AR: abnormal readings; cf-DNA: circulating cell-free DNA; CPAP: continuous positive pressure airway; DBP: Diastolic Blood Pressure; FMD: Flow mediated dilatation; MPs: microparticles; PP: pulse pressure; SBP: Systolic Blood Pressure; VEGF: Vascular endothelial growth factor.

At the same time, the endothelial function assessed by FMD by Laser-Doppler flowmetry improved, since the area of hyperaemia after the ischemia significantly increased. Moreover, markers of endothelial damage (CD31^+^/annexin V^+^ MPs and circulating cf-DNA) decreased, whereas a marker of reendotelialization (VGEF) increased.

**Fig 1:**
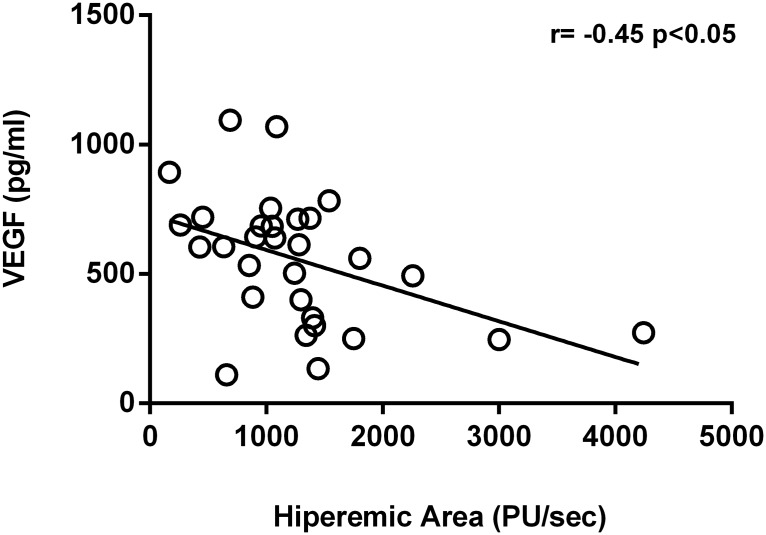
Relationship between flow mediated dilatation measured by Laser-Doppler flowmetry (hyperaemic area) and vascular endothelial growth factor (VEGF).

Fig 1 shows how the hyperaemic area after ischemia was negatively related to VEGF. However FMD did not correlate with CD31^+^/annexin V^+^ MPs or circulating cf-DNA. BP levels did not correlate with VEGF. CD31^+^/annexin V^+^ MPs and circulating cf-DNA correlated with 24-h diastolic BP (r = 0.39, p<0.05, and r = 0.41, p<0.05, respectively), daytime diastolic BP (r = 0.38, p<0.05; r = 0.41, p<0.05), 24-h mean BP (r = 0.43, p<0.01; r = 0.39, p<0.05) and daytime mean BP (r = 0.44, p<0.01; r = 0.39, p<0.05). 24-h systolic BP also correlated with circulating cf-DNA (r = 0.42, p<0.01).

**Fig 2:**
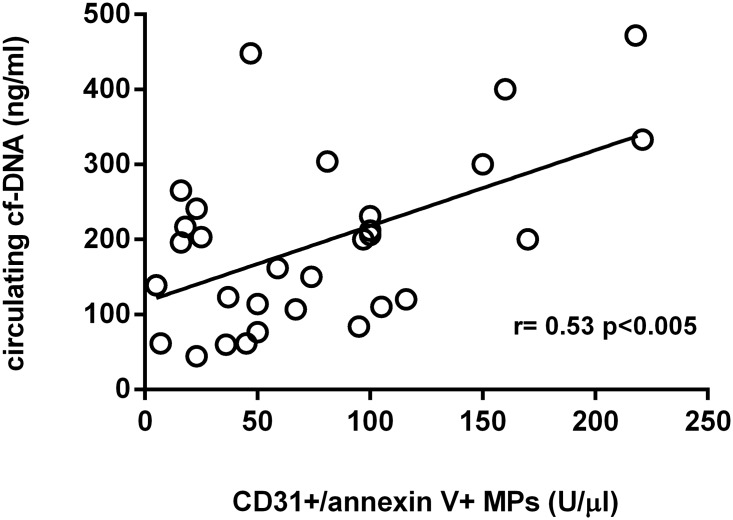
Relationship between CD31^+^/annexin V^+^ MPs and circulating cf-DNA.

As it is observed in the Fig 2, both markers of endothelial damage are positively related each other. However, there was not any significant relationship between CD31^+^/annexin V^+^ MPs and VEGF (r = -0.27, p = 0.25) or between circulating cf-DNA and VEGF (r = 0.02, p = 0.93).

**Fig 3:**
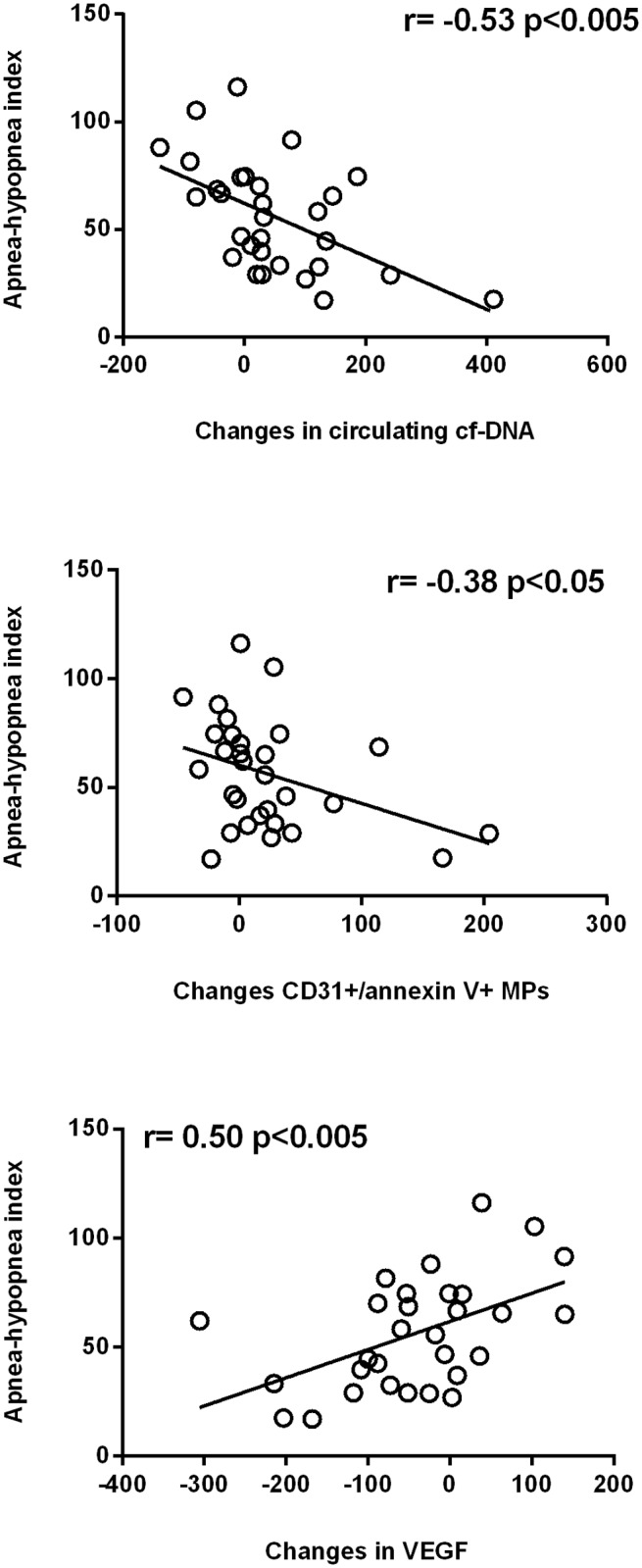
Relationship among the severity of the disease measured according to the apnoea-hypopnea index and changes from baseline in circulating cf-DNA, CD31^+^/annexin V^+^ MPs and vascular endothelial growth factor (VEGF).

**Fig 4:**
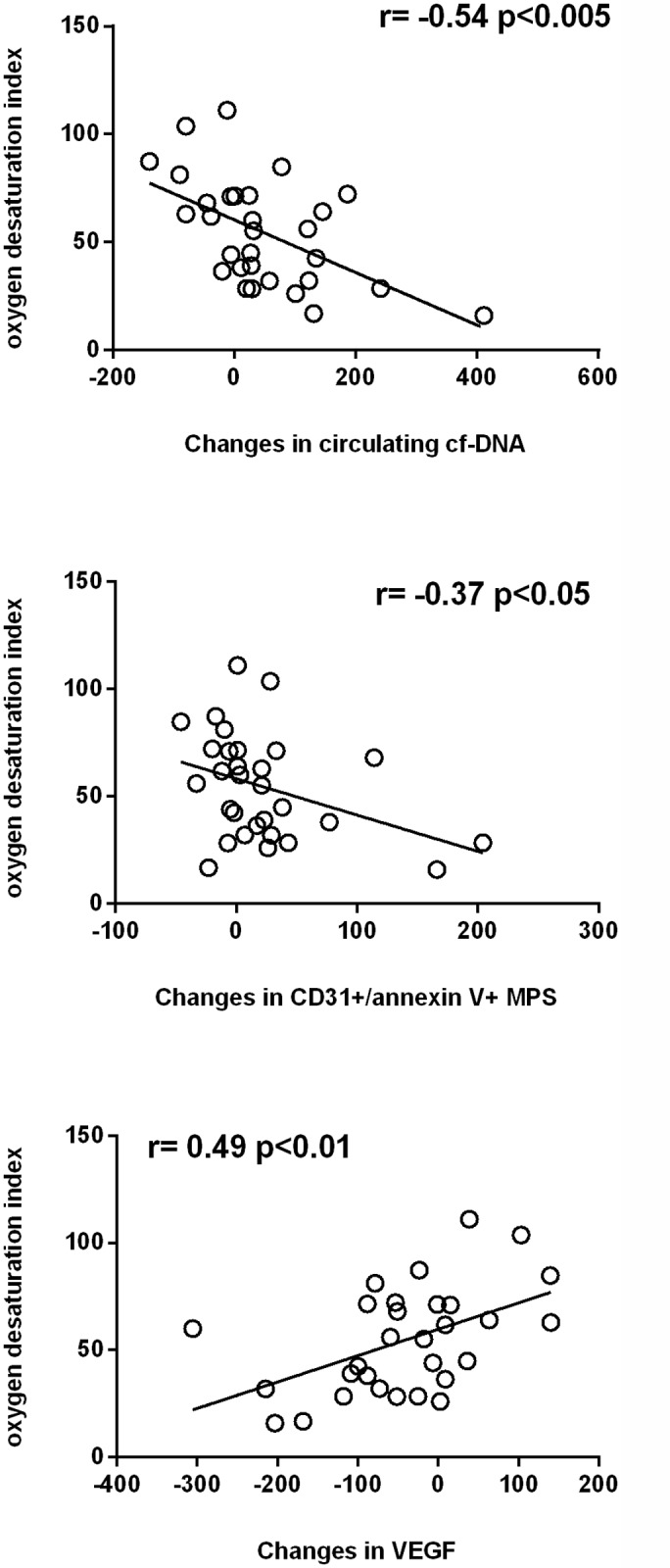
Relationship among the severity of the disease measured according to the oxygen desaturation index, and changes from baseline in circulating cf-DNA, CD31^+^/annexin V^+^ MPs and vascular endothelial growth factor (VEGF).

**Fig 5:**
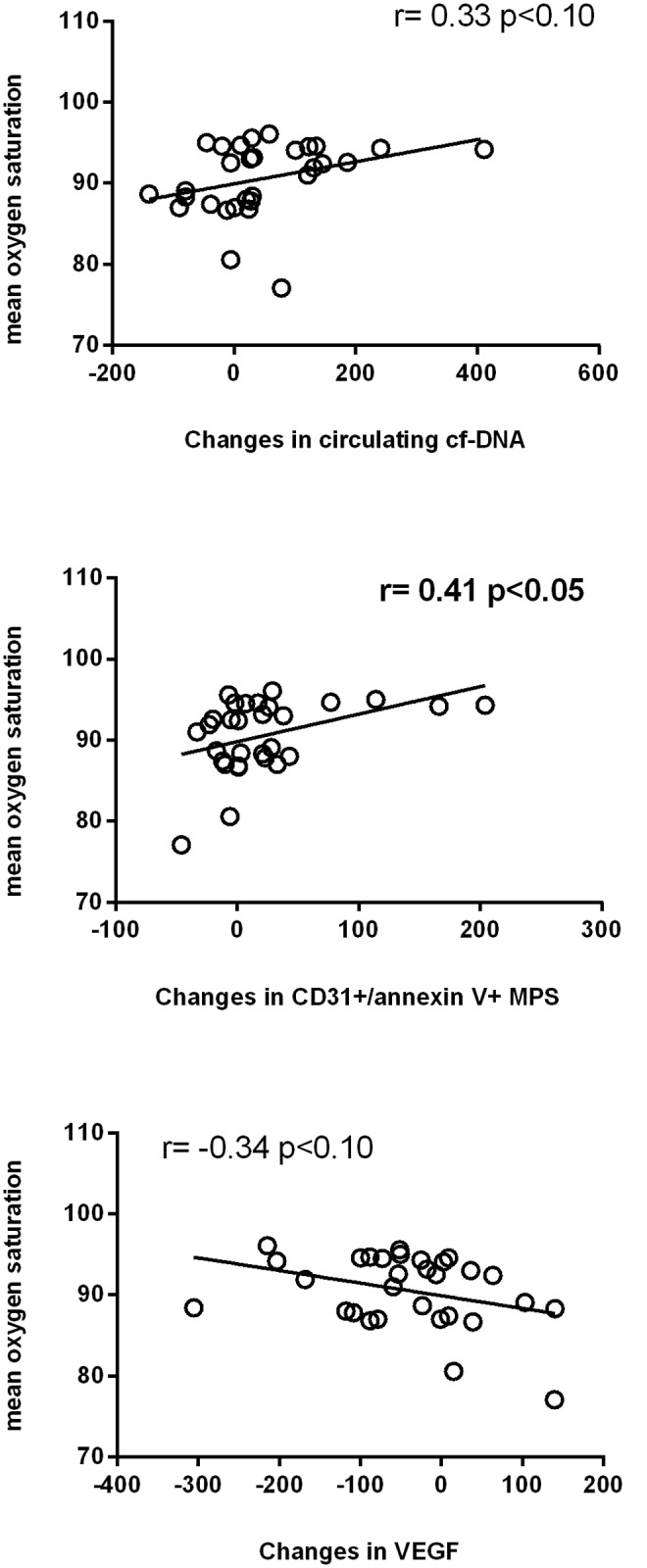
Relationship among the severity of the disease measured according to the mean oxygen saturation (%), and changes from baseline in circulating cf-DNA, CD31^+^/annexin V^+^ MPs and vascular endothelial growth factor (VEGF).

Figs 3 and 4 show how the apnoea-hypopnea index and the oxygen desaturation index, respectively, were correlated inversely with the changes observed in the values of circulating cf-DNA or CD31^+^/annexin V^+^ MPs and positively with the changes in VEGF levels. Conversely, Fig 5 displays mean SaO2 correlating positively with the changes observed in cf-DNA or CD31^+^/annexin V^+^ MPs and correlating inversely with changes in VEGF values (although the last correlation did not reach statistical significance (p<0.10).

## Discussion

Vascular function has been assessed *in vivo* using the FMD on brachial artery with ultrasounds. However, more recently a new technique has appeared for measuring FMD using Laser-Doppler flowmetry. This technique is more observer-independent and it measures microcirculation rather than vascular function in higher arteries. As far as we know, there are few studies with this technique in patients with OSA, and only one of them evaluating the effect of CPAP on FMD [15].

In the present study we have observed a marked improvement in FMD (expressed as the increment in the hyperemia area measured in PU) after CPAP therapy (Table 2). This improvement was not related to the two markers of endothelium damage that we measured (CD31^+^/annexin V^+^ MPs and circulating cf-DNA); however, it was negatively related to a marker of restoration of the endothelium (VEGF) (Fig 1), perhaps indicating that patients with a good endothelial function do not need to have high values of VEGF and viceversa. However, the influence of additional factors that may confuse this relationship cannot be ruled out; for instance, the time of exposure to the sleep apnea: a shorter time may lead to lesser impairment of endothelial function and lower VGEF levels. Nevertheless, patients were all included soon just after the diagnosis, to minimize differences among patients in this regard.

MPs are small (<1 μm) vesicles that are released from the endothelium in response to several injuries. The level of circulating MPs in peripheral blood has been reported to be increased in several ischemic diseases [7]. It seems that MPs from activated leukocytes (CD62L_ MPs) are higher in patients with OSA and a positive correlation between circulating levels of CD62L_ MPs and nocturnal hypoxemia severity has been reported [16,17,18]. Nevertheless, other authors studying children with sleep breathing disorders observed that leukocyte CD11b+ MPs and platelet CD41a+ MPs were the microparticles that correlated the best with the apnoea-hypopnea index [19].

In our study we measure CD31^+^/annexin V^+^ MPs (microparticles marked with anti-CD31 antibody, followed by incubation with annexin V) in plasma and we found a significant decrease after CPAP therapy (Table 2), which was more evident in patients with a more severe disease (Figs 3–5). We chose these types of CD31^+^/annexin V^+^ MPs because they have been found to be increased in other cardiovascular pathologies [10, 20, 21] and, to our best knowledge, they have never been investigated in OSA syndrome. In our work we also found that this type of CD31^+^/annexin V^+^ MPs was well related to other markers of endothelial dysfunction, such as circulating cf-DNA (Fig 2).

On the other hand, it has also been observed that cf-DNA levels rise in pathologies involving ischemia, such as acute coronary syndrome, ischemic heart failure, stroke, mesenteric ischemia or in patients who have suffered a cardiac arrest outside the hospital [22,23]; we have also recently reported higher levels of this marker of endothelial damage in preeclampsia and HELLP syndrome [11]. These both pathologies have some similarities with the OSA syndrome, since in both conditions the hypoxia (systemic or specific organ-related) can play a role (the origin of the aforementioned pathologies has been suggested to be placental hypoxia) and patients can be studied during the pathologic state as well as after the improvement of the intermittent hypoxia (OSA syndrome) or the recovery of the disease (after delivery, when the placenta is removed). The interesting results obtained in that study encouraged us to measure cf-DNA in patients with OSA syndrome, before and after CPAP therapy. Our results indicate that the cf-DNA levels drop after CPAP and that this decrease is higher in those patients with a more severe disease (Table 2, Figs 3–5). These results are in agreement with the previously communicated by Ye et al [24] where cf-DNA correlated positively with AHI and oxygen desaturation index, negatively with lower SaO2; and, as occurs in our case, they decreased after 6 month of CPAP therapy. Up to our best knowledge, the work from Ye et al and our study are the only studies who have measured cf-DNA in OSA syndrome.

Remarkable findings in our study are that 1) plasma values of VEGF rises after CPAP, with a higher increase in those patients with a more severe disease, and 2) the baseline levels of VEGF were negatively related to FMD, thus suggesting that patients with a better endothelial function may need lesser VEGF levels. In a previous study in 2001, Imagawa et al [25] described that VEGF values were higher in patients with OSA syndrome compared with controls, with a steadily increase according to the AHI. In agreement with this, Kaczmarek et al [26] demonstrated a significant upregulation of VEGF expression in skin biopsies obtained from OSA patients with severe nocturnal hypoxemia (SaO2 <75%) compared with mildly hypoxemic OSA patients (SaO2 75%-90%). Nevertheless, another study comparing patients with an AHI >15/h, subjects with an AHI <5/h and patients with OSA on CPAP treatment, did not find significant differences among groups [27]. Similarly, other authors observed that withdrawal from CPAP therapy was not accompanied by any change in plasma values of VEGF [28].

To our knowledge, our study is the first that considers VEGF, CD31^+^/annexin V^+^ MPs and cf-DNA together. Despite its results and contributions, we must acknowledge some limitations. For instance, the small number of participants, the absence of a control group or the assessment of the markers after withdrawal of CPAP, that would have allowed a better assessment of the behaviour of the endothelial markers. A longer follow-up may have also demonstrated additional changes due to more chronic exposure to CPAP therapy. Notwithstanding the previous, we must highlight that we have found significant changes and correlations between the endothelial markers and, remarkably, these changes occurred in the short-medium term (3-month CPAP therapy), suggesting a rapid beneficial action of CPAP on the endothelium mediated by the attenuation of the hypoxia-related damage. Finally, we do not have a clear explanation why there was not any significant relationship between markers of damage and vascular development. The heterogeneity of the subjects (e.g.: subjects with high both damage and restorations while others with high restoration and low damage or viceversa) might be a reason to consider. This fact could support the necessity of studying both types of markers at the same time.
